# Genomic information and a person’s right not to know: A closer look at variations in hypothetical informational preferences in a German sample

**DOI:** 10.1371/journal.pone.0198249

**Published:** 2018-06-20

**Authors:** Laura Flatau, Markus Reitt, Gunnar Duttge, Christian Lenk, Barbara Zoll, Wolfgang Poser, Alexandra Weber, Urs Heilbronner, Marcella Rietschel, Jana Strohmaier, Rebekka Kesberg, Jonas Nagel, Thomas G. Schulze

**Affiliations:** 1 Institute of Psychiatric Phenomics and Genomics, Ludwig-Maximilians-University, Munich, Germany; 2 Institute of Psychiatry and Psychotherapy, University of Göttingen, Göttingen, Germany; 3 Center for Medical Law, University of Göttingen, Göttingen, Germany; 4 Institute for History, Theory and Ethics of Medicine, Ulm University, Ulm, Germany; 5 Institute for Human Genetics, University of Göttingen, Göttingen, Germany; 6 Central Institute for Mental Health, Mannheim, Germany; 7 Institute for Psychology and Education, Ulm University, Ulm, Germany; 8 Department of Psychology, University of Göttingen, Göttingen, Germany; University of Utah, UNITED STATES

## Abstract

In clinical practice and in research, there is an ongoing debate on how to return incidental and secondary findings of genetic tests to patients and research participants. Previous investigations have found that most of the people most of the time are in favor of full disclosure of results. Yet, the option to reject disclosure, based on the so-called right not to know, can be valuable especially for some vulnerable subgroups of recipients. In the present study we investigated variations in informational preferences in the context of genetic testing in a large and diverse German sample. This survey examined health care professionals, patients, participants of genetic counseling sessions and members of the general population (N = 518). Survey participants were assessed regarding their openness to learning about findings under various hypothetical scenarios, as well as their attitudes about the doctor-patient-relationship in a disclosure situation and about informational transfer to third parties. While the majority of participants wanted to learn about their findings, the extent of support of disclosure varied with features of the hypothetical diagnostic scenarios (e.g., controllability of disease; abstract vs. concrete scenario description) and demographic characteristics of the subjects. For example, subjects with higher levels of education were more selective with regards to the kind of information they want to receive than those with lower levels of education. We discuss implications of these findings for the debate about the right not to know and for the clinical practice of informed consent procedures.

## Introduction

The rapidly increasing use of high throughput genome sequencing techniques in medicine is accompanied by a similarly increasing number of ethical and juridical questions. Of particular importance is the implementation of high throughput procedures in clinical practice [1,2], where it can be used for various purposes (e.g., to individualize treatment by predicting a patient’s tolerance of different medication options). Nowadays, the entire human genome can be sequenced and analyzed rapidly at ever-decreasing costs. Such high-throughput methods are very likely to produce incidental findings [3]. The American College of Medical Genetics and Genomics (ACMG) defines incidental or additional findings as “…results of a deliberate search for pathogenic or likely pathogenic alterations in genes that are not apparently relevant to a diagnostic indication for which the sequencing test was ordered […] but that may nonetheless be of medical value or utility to the ordering physician and the patient” [4]. Secondary findings are defined as findings that are “actively sought by a practitioner that is not the primary target” (Presidential Commission for the Study of Bioethical Issues, 2013).

Incidental or secondary findings bear the potential for ethical and psychological conflict, as the disclosure of unexpected and potentially disturbing information can lead to considerable psychological discomfort. The obligation of healthcare professionals to fully inform patients about their health status might clash with the patients’ right to informational self-determination, involving the so-called “right not to know” which grants people the right not to be confronted with unwanted information about their personal matters [5]. An important question is thus how practitioners should deal with the existence of such additional information.

Ethicists, clinicians and lawyers offer answers and recommendations that range from never disclosing anything that was not intended, to disclosing every finding [6–9]. Central issues in this discussion are the clinical and personal utility of incidental or secondary findings. Potential positive consequences, e.g. behavioral changes benefitting health [10], and negative consequences, e.g. psychological harm [11,12], of a disclosure of incidental findings have been discussed. Furthermore, the ongoing debate tries to formulate different rules and procedures for contexts with different goals (e.g., research vs. clinical) [13]. Regarding the research context, Ramoni et al. found that just a few investigators (about 4%) had experience in returning individual results, but that the majority stated that return of results is appropriate under at least some circumstances [14]. Evans and Rothschild argued for a minimum obligation to disclose incidental findings in research [15]. Concerning the clinical setting, in 2013 the ACMG provided a minimum list of 56 genetic mutations which are actionable that must be disclosed in the clinical setting, regardless of the patient’s preference [4]. In consequence of critical reactions (Presidential Commission for the Study of Bioethical Issues, 2013) the statement was revised in 2016 to a list including “59 medically actionable genes recommended for return in clinical genomic sequencing”, which are no longer mandatory [16]. In 2004, the German Federal Parliament implemented the ‘right not to know’ in the law and described it as manifestation of the right of informational self-determination (Genetic Diagnosis Act, 2004). In sum, despite considerable controversy about its adequate implementation and its limits, the general importance of the right not to know is not under dispute today.

### Informational preferences in the context of genetic testing

The necessity to formulate a right not to know arises when people prefer ignorance over full information about their personal matters. When and to what extent people have such a preference for ignorance are empirical questions that can be assessed with methods from the social sciences. Even though data on people’s informational preferences cannot tell us directly which social regulations we should implement (“no ought from an is”), they might nonetheless provide fruitful input for the normative debate outlined above. For example, they might serve to estimate the subjective importance of the existence of a right not to know for different groups of people, or to delineate the conditions under which a failure to enforce such a right might be especially consequential.

Recent research in cognitive psychology suggests that people often prefer ignorance over full information [17–19]. For example, a recent study found that 85–90% would not want to know in advance which negative events would strike them in the future (e.g., cause of death, divorce) [17]. However, the preference for ignorance with regards to potentially threatening upcoming life events seems to be less pronounced in the context of genetic testing. Several studies demonstrate that genetic testing is generally evaluated positively in the public [20]. It has been shown that the majority wants to learn about their results and that there is almost no difference between risk information (e.g., information about carrier status) and information about a secured diagnosis (e.g., onset of dementia) [21–26]. Whereas experts emphasize clinical utility as decision criterion for disclosure [27], participants mention that they would also personally benefit from genetic information without clinical relevance [28–30]. Analyzing time trends within public attitudes, a comparison between 2002 and 2010 showed that people in 2010 anticipated more use of genetic information and were more interested in their genetic make-up, but also expected more social discrimination in the context of genetic testing compared to participants in 2002 [31].

Despite this general picture, some studies have discovered important moderators for people’s interest in learning about their results. As can be expected, this interest seems to drop considerably when the results concern uncontrollable as compared to preventable diseases. For instance, 88% of a Canadian sample with patients who were at high risk for developing Huntington’s disease did not participate in a predictive test [32]. Yaniv and Sagi used hypothetical scenarios and found that about 50% of their participants did not want to receive genomic information about their healthcare status regarding Huntington’s disease. As reasons for not wanting to know participants mentioned ‘lack of treatment’ and ‘anxiety, depression and stress’ [33]. Melnyk and Shepperd showed that lacking coping resources, anticipated regret und reading about uncontrollable predictors are associated with avoidance of risk information about breast cancer [34]. In another study, the concrete decision on wanting genomic information in hypothetical scenarios presenting cases of devastating late-onset diseases was predictable through explicit features of the disease scenario, namely the ‘controllability of the disease’ and the ‘power of the test’ [35].

Some studies found additional moderators next to controllability. Henneman and colleagues surveyed the attitudes towards genetic testing of a Dutch sample and identified opponents (30%) and supporters (32%) of genetic testing. Being a supporter was related to the belief in benefits of testing and being confident that genetic information might help in establishing a healthy lifestyle. Opponents were more likely to believe that genetic testing is tampering with nature. Demographic variables as level of education, gender and age were not associated with being opponent or supporter [36]. In a Russian sample 85% of 2000 participants were interested to undergo predictive genetic testing for preventable (controllable) health conditions. The factors most strongly related to a high level of interest were willingness to improve one’s lifestyle and overestimated expectation towards genetic testing [37]. A survey in Finland showed that the majority approved of genetic tests and stressed positive consequences. Interestingly, participants with the highest level of genetic knowledge took more extreme positions (enthusiasm and skepticism) [38].

In sum, the general picture of the previous findings depicts high levels of interest in learning about potentially disturbing results of genetic tests, but that there are also important boundary conditions that significantly reduce openness to such information. In the present study, our main aim is to broaden the database on people’s informational preferences in the context of genetic testing by investigating a large and diverse German sample. We focus on the openness towards the receipt of genetic findings in different hypothetical diagnostic scenarios with varied availability of prevention and/or intervention options (e.g. breast cancer, Chorea Huntington). Furthermore, we investigate preferences regarding doctors’ behavior in a disclosure situation and informational transfer to third parties (e.g. insurance companies). Subsequently, we take an exploratory look at demographic characteristics that might be associated with informational preferences regarding the disclosure of incidental or secondary findings. Previous research indicates that prior experience with genetic tests (either as patient or healthcare professional) might be especially important [9,25,27,38,39]. In addition, we conducted exploratory analyses of how our participants’ gender and educational level relates to their attitudes. We conclude by discussing the implications of our findings for the practice of pre-test counseling and by outlining open questions for future research.

## Materials and methods

### Participants and assessment procedure

523 participants completed the questionnaire assessing the ‘normative foundation and practical validity of the right not to know’ (*www.recht-auf-nichtwissen.uni-goettingen.de*). The questions were developed in discussions with experts of the field of genetic, psychiatric and juridical research and based on literature research. A pilot study including an interview with a human geneticist unrelated to the project was conducted to ensure the comprehensibility and quality of the questionnaire. The questionnaire was approved by the ethics committee of the University of Göttingen Medical Center (reference number 20/1/13; for further information about the approval process, see *www.ethikkommission.med.uni-goettingen.de*). The final version of the questionnaire consists of 53 items (see S1 File).The survey was conducted via a paper-pencil (*N* = 335) and an online-version (*N* = 188). Paper-pencil questionnaires were mainly completed by individuals seeking genetic counseling, patients, and healthcare professionals at the University of Göttingen Medical Center. The material was distributed by providing questionnaires in the clinic’s waiting rooms or at notice-boards in the area of the Medical Center of the University of Göttingen. Interested participants were encouraged to send completed questionnaires back to the Institute of Medical Law or to directly transfer it to medical staff. Additional questionnaires were distributed in other locations in Göttingen (e.g. administrative bodies, resident physicians, notice-boards, etc.). The online survey was published via newsletters and homepages (e.g. German Association for Bipolar Disorder (DGBS), The German Association for Psychiatry, Psychotherapy and Psychosomatics (DGPPN), University of Göttingen Medical Center). Data were collected from June 2014 to November 2014. No personally identifying data were collected, and the participants took part in the survey voluntarily. Five participants (three paper-pencil, two online) reported an age under 18 years and were therefore excluded from the analyses. Characteristics of the remaining sample (*N* = 518) are summarized in Table 1. Comparing the demographic variables with regard to the recruitment procedure, the online version reached more highly educated subjects and more subjects with a professional role in the health care system than the paper-and-pencil version.

**Table 1:** Descriptive measures of the sample.

Demographic variable		Descriptive statistics (*N* = 518)
Age		*M* = 42.5, *SD* = 13.9 (*range* 18–77)
Sex		
	Male	30.6% (*N* = 147)
	Female	69.4% (*N* = 334)
Educational level		
	12–13 years of school	55.9% (*N* = 279)
	10 years of school	25.5% (*N* = 127)
	≤9 years of school	15.0% (*N* = 75)
	Other	3.6% (*N* = 18)
Religion		
	Catholic	22.9% (*N* = 115)
	Protestant	40.4% (*N* = 203)
	No confession	32.0% (*N* = 161)
	Other	4.8% (*N* = 24)
Professional role in the healthcare system		
	Physician	7.3% (*N* = 35)
	Nurse	4.4% (*N* = 21)
	Medical student	2.5% (*N* = 12)
	Other	21.0% (*N* = 101)
	None	64.8% (*N* = 311)
Level of being affected by somatic genetic disorder		
	Themselves	13.8% (*N* = 71)
	Family members	23.0% (*N* = 119)
	Themselves and family members	11.1% (*N* = 57)
	Not affected	52.0% (*N* = 268)
Affected by psychiatric disorder		13.9% (*N* = 72)

### Instruments

The questionnaire consisted of 53 multiple-choice items and examined participants’ attitudes towards different areas concerning medical findings. First, demographic variables were collected. Participants were asked about their role in the healthcare-system and to what extent they or their family members suffered from genetic or other severe diseases (e.g. psychiatric disease). In the subsequent sections, the participants were asked about their general attitudes towards incidental and secondary findings and the use of personal health data. This section was followed by hypothetical diagnostic scenarios in which clinically relevant findings were presented and participants could decide if they are in favor of learning this information or not. We varied the disorder (e.g. dementia, breast cancer), the consequences of having that disorder (e.g. wheel chair, morbidity), age and certainty of outbreak (e.g. risk information), context of disclosure (e.g. clinical vs. research context), and availability of treatment or prevention. The next section examined attitudes towards the doctor-patient relationship, focusing on the particular ethical concepts of autonomy and duty of care. Participants were asked for their preferences concerning the doctor´s behavior in the case of an incidental or secondary finding (e.g. if it is reasonable to override someone´s wish not to know in case that a severe and actionable finding has been found). The final section asked about attitudes towards diagnostic scenarios involving third parties (e.g. offspring, other relatives, or insurance companies). The questions focus on the openness towards information transfer to third parties and on perceived obligations of others to conduct genetic tests (e.g. pilots, partners). Parts of the questionnaire were based on the GenEthics Questionnaire and modified by members of the BMBF project group “Normative fundament of the right not to know” [16]. For the detailed questionnaire, see supporting information (S1 File).

### Statistical analysis

Statistical analyses were performed with the software package SPSS (IBM Corp. Released 2013. IBM SPSS Statistics for Windows, Version 22.0., Armonk, NY: IBM Corp). To compare attitudes of different groups (e.g. professionals vs. general population) chi-square analyses were carried out. Items that were assessed with a continuous scale were dichotomized according to whether participants qualitatively indicated agreement or disagreement prior to analysis. Indications of indifference were assigned to the “I don’t know” category.

## Results

The results section is structured as follows. In the first sub-section we present descriptive results regarding informational preferences across the entire sample to convey a descriptive impression of the attitudes of our subjects. We follow the structure of the questionnaire by displaying results concerning the wish to know or not to know, the attitudes towards the doctor-and patient relationship, and the preferences regarding informational transfer to third parties. In the second sub-section we take a closer look at the demographic factors potentially associated with informational preferences (being affected personally, gender, educational level, professional role in the healthcare system).

### Informational preferences across the entire sample

#### The wish to know

Ninety-one percent of our participants agreed with the statement that everybody had the right to know everything about her or his genetic disposition including risks and carrier status information for genetic diseases. Eighty-two percent wanted to learn about every incidentally found disease. In case of an incidentally found risk of a genetic disease, 66% wanted to be informed about that. Forty-eight percent would participate in a genetic test for 250 potential disorders, if there were an accessible and affordable option to test. When a specific description of disease and possible ways of intervention or/and prevention were presented, 80% of the respondents wanted to know if they had a genetic precondition for heart attack (no special intervention available). Disclosure of information about a precondition for breast cancer with options of intervention and prevention was favored by 88% of the participants. When the scenario shifted to various types of cancer without possibilities of prevention, this number dropped to 69%. Receiving information about a genetic risk for myatrophy by the age of 30 to 40 was welcomed by 74%.

In summary, the results show that the majority of the participants wanted to be informed about potential findings regarding genetic diseases. Despite this openness to information, 75% agreed with the statement that they would expect to experience emotional distress upon learning about these conditions. Furthermore, 51% thought that knowledge of these findings could lead to societal discrimination.

#### The wish not to know

**Fig 1:**
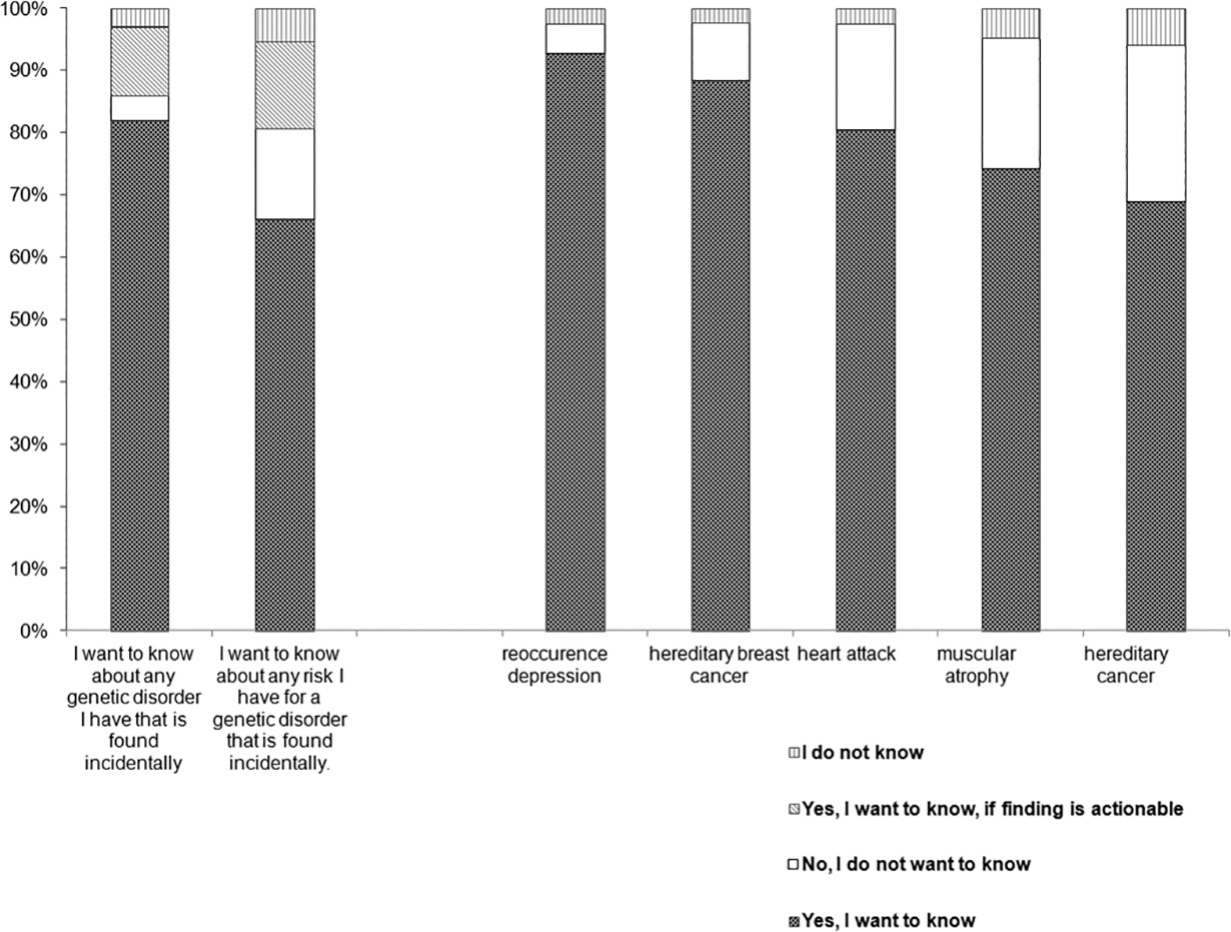
The wish to know in different diagnostic scenarios.

Being asked about unspecified findings in an abstract way (without describing a concrete scenario; e.g., “I want to know about any (genetic) disorder I have that is found incidentally”), 3–4% rejected the disclosure of information. When we asked about the wish to receive risk information 14% answered they would reject that information. In concrete scenarios with mention of different findings including diagnosis, consequences, and intervention possibilities, 5% to 25% decided to reject the disclosure of those findings (see Fig 1).

Answers differed with respect to different diagnoses. In case of a risk for reoccurrence of a depressive episode (i.e., after having suffered from depression before), 5% would reject the disclosure, whereas disclosure about a genetic predisposition to cancer would not be welcomed by 25% (further examples: dementia: 10%; heart attack: 17%, breast cancer: 9%, myatrophy: 21%).

#### Doctor-patient-relationship with respect to the wish not to know

The third section examined attitudes towards the extent of the physician’s responsibilities with respect to the wish to know and not to know. 35% of the respondents agreed with the statement that their physician should know everything about their genetic health condition and decide him- or herself which information is disclosed. 68% were of the opinion that, if they had to decide between the ethical principles of the respect for patient´s autonomy and the physician duty of care, their autonomy outweighed the medical duty of care. Being asked if the physician should have the right to ignore the patient’s decision to remain ignorant, 66% were against this type of behavior and 21% would tolerate that decision only in exceptional cases. These exceptional cases included cases of life and death and consequences for others.

#### Third parties

The fourth section examined attitudes toward a potential involvement of third parties, especially information transfer of a finding. Whereas 85% considered informing their relatives in case of receiving results that could also affect family members, only 58% agreed with the statement that they would always want to be informed if their relatives were affected. Ninety-two percent did not want their insurance companies to have the right to examine their genetic predisposition. Being asked if individuals with high responsibilities in their job (e.g. pilots) should have a mandatory genetic sequencing test, 32% approved this idea. Fifty-five percent of the respondents did not consider it necessary to test their partners with regard to reproductive concerns. Thirty-seven percent did not want to test themselves in order to check their reproductive conditions. Thirty-nine percent wished to know if their children had a genetic precondition for a disease during pregnancy.

#### Demographic factors associated with informational preferences

In a next step, we investigated how demographic and phenotypic features (e.g. educational level, gender) were related to participants`informational preferences (e.g. towards full disclosure).

#### Affected versus unaffected individuals

Affected individuals (people with genetic disease or relatives of ill people) did virtually not differ in any way from non-affected individuals in our sample.

#### Gender

Of the male participants, 68% revealed that they would take a genetic test to examine their reproductive conditions whereas only 50% of female participants would take that test (χ2 (2, N = 461) = 13.5, *p* < .001). Additionally, men were slightly more motivated to enroll in a sequencing test for 250 genetic diseases than women (56% of the men and 44% of the woman agreed, χ2 (2, N = 466) = 5.9, *p* < .05).

#### Educational level

The data revealed differences on many items with regard to the educational level (EL). Detailed information about percentages of agreement split up by EL is displayed in Table 2. Respondents with a higher EL (12 to 13 years of school, corresponding to higher education entrance qualification) were significantly more likely to emphasize the patient’s autonomy over the physician’s responsibility than participants with a low average EL (≤10 years of school) (χ2 (2, N = 480) = 10.3, *p* < .01). In case of a severe disease without possibilities for therapy, respondents with high EL were more likely to disapprove of the physician’s disrespect for an agreed-upon non-disclosure than low-EL respondents (χ2 (2, N = 474) = 48.0, *p* < .001). People with low EL wanted their doctor to know and decide everything regarding their physical condition to a greater extent than participants with higher EL (χ2 (2, N = 475) = 27.2, *p* < .001).

**Table 2:** Percent agreement by educational level for selected items.

Response Options	Educational Level		Statistics
	12–13 years	≤10 years	
Do you want to know if you inherited the genetic predisposition for cardiovascular disease?			
yes	76%	85%	*χ2* (2, N = 481) = 19.0, *p* < .001
no	23%	10%	
I don’t know	1%	5%	
Do you want to know if you have inherited the genetic makeup for hereditary cancer?			
yes	65%	73%	*χ2* (2, N = 484) = 11.8, *p* < .01
no	31%	19%	
I don’t know	4%	8%	
My physician should know all my genetic findings and decide on the basis of his professional knowledge which he tells me about and which he doesn’t tell me about.			
yes	25%	46%	*χ2* (2, N = 475) = 27.2, *p <* .001
no	73%	50%	
I don’t know	2%	5%	
Which of the following do you think outweighs the other: The physician’s duty of care towards you as a patient or your right to self-determination?			
Duty of care	16%	27%	*χ2* (2, N = 480) = 10.3, *p <* .01
Self-determination	74%	61%	
I don’t know	10%	12%	
Physician overrides your decision not to know in case of risk information about a non-actionable severe disease. Do you agree?			
Yes, I agree	30%	57%	*χ2* (2, N = 474) = 48.0, *p <* .001
No, I do not agree	61%	29%	
I don’t know	10%	14%	
Would you want to know already before the birth whether your child has a genetic risk for a genetic disorder?			
yes	34%	45%	*χ2* (2, N = 479) = 9.7, *p <* .01
no	60%	46%	
I don’t know	6%	9%	
Should people who have jobs with special responsibility (e.g. pilots) be tested for certain genetic risks?			
yes	25%	41%	*χ2* (2, N = 483) = 31.7, *p* < .001
no	67%	42%	
I don’t know	8%	17%	
I want to know about any risk I have for a genetic disorder that is found incidentally.			
yes	57%	77%	*χ2* (3, N = 482) = 20.6, *p* < .001
no	19%	11%	
Only, if prevention possibilities are available	17%	9%	
I don’t know	7%	3%	
Depending on the following age of onset of the illness, would you want to know if you have the genetic defect? 20 Years			
yes	77%	72%	*χ2* (2, N = 443) = 5.3, *p* = .071
no	19%	18%	
I don’t know	5%	10%	
Depending on the following age of onset of the illness, would you want to know if you have the genetic defect? 40 Years			
yes	78%	82%	*χ2* (2, N = 458) = 8.0, *p* < .01
no	18%	10%	
I don’t know	4%	8%	
Depending on the following age of onset of the illness, would you want to know if you have the genetic defect? 60 Years.			
yes	63%	71%	*χ2* (2, N = 465) = 21.0, *p <* .*001*
no	33%	17%	
I don’t know	4%	12%	

*Note*. For reasons of space, the wording of the questions displayed in this table is abbreviated. Please see S1 File for the exact question wording in the original questionnaire. Percentages might not add up to 100% due to rounding errors. All reported *p*-values are Bonferroni corrected.

The wish to know everything regarding risk factors was significantly less pronounced in people with high EL (χ2 (3, N = 482) = 20.6, *p* < .001). In scenarios where a concrete case is defined, including potential consequences of the disease, people with higher EL were more likely to reject the disclosure (case hereditary cancer: χ2 (2, N = 484) = 11.8, *p* = <0.01; case cardiovascular disease: χ2 (2, N = 481) = 19.0, *p* < .001). In the group of the people with high EL, there were significantly more people who did not want their offspring to be tested (χ2 (2, N = 479) = 9.7, *p* < .01), nor people with special responsibilities (e.g. pilots; χ2 (2, N = 483) = 31.7, *p* < .001).

We also asked patients to imagine they were 18 years of age and confronted them with findings of a genetic disease with various ages at disease onset (20 years, 40 years, or 60 years). In participants with high EL, age at onset had an impact on their desire to learn about their genetic risk. The later the disease onset, the less highly educated people wanted to know and the more they could be distinguished from people with low EL (onset at 20 years: χ2 (2, N = 443) = 5, *p* = .071; onset at 40 years: χ2 (2, N = 458) = 8.0, *p* < .01, onset at 60 years: χ2 (2, N = 465) = 21.0, *p* < .001). In summary, highly educated people had a tendency to emphasize the patient’s autonomy and were more likely to reject information resulting from genome sequencing than less educated people.

#### Professional role in the healthcare system

Detailed information about percentages of agreement regarding professional role in the healthcare system is displayed in Table 3. Professionals rejected disclosure of a hypothetical incidental or secondary finding significantly more often than participants without such professional background (information about any disease: χ2 (9, N = 462) = 17.0, *p* < .05). They objected to receiving a finding or information on risk more often (χ2 (9, N = 463) = 25.7, *p* = < .01) and they were less willing to participate voluntarily in an inexpensive genetic test (χ2 (6, N = 464) = 15.5, *p* < .05). Furthermore, they tended to emphasize the patient’s autonomy in health decisions as opposed to the view that the physician knows best and should make decisions in a patient’s favor (χ2 (6, N = 461) = 13.2, *p* < .05). Also, physicians and medical students were significantly less willing to have themselves or their children tested in order to examine their reproductive conditions as compared to participants without a role in the healthcare system (χ2 (6, N = 459) = 16.0, *p* < .01). In sum, physicians and medical students tended to indicate less interest in medical information disclosure compared to people outside the health care system.

**Table 3:** Percent agreement by professional role in the healthcare system for selected items.

Response Options	Professional Role				Statistics
	Physicians/ Medical Students	Nurses	Other Role	No Role	
I want to know about any risk I have for a genetic disorder that is found incidentally.					
yes	37%	84%	61%	70%	*χ2* (9, N = 463) = 25.7, *p* = < .01)
no	28%	5%	16%	12%	
Only, if prevention possibilities are available	24%	11%	14%	14%	
I do not know	11%	0%	9%	4%	
I want to know about any disease I have that is found incidentally.					
yes	65%	81%	81%	85%	*χ2* (9, N = 462) = 17.0, *p* < .05)
no	4%	0%	4%	3%	
Only, if prevention possibilities are available	24%	14%	10%	11%	
I do not know	7%	5%	5%	1%	
There is a simple and reasonably priced option to be tested for your risk for more than 250 genetic disorders. Would you get yourself tested?					
yes	32%	40%	45%	52%	*χ2* (6, N = 464) = 15.5, *p* < .05)
no	60%	40%	47%	34%	
I do not know	9%	20%	8%	13%	
Would you have yourself genetically tested so you can better assess the risk that (future) children will develop a serious disease?					
yes	50%	55%	50%	57%	*χ2* (6, N = 459) = 16.0, *p* < .01)
no	48%	20%	44%	36%	
I do not know	2%	25%	5%	7%	
My physician should know all my genetic findings and decide on the basis of his professional knowledge which he tells me about and which he doesn’t tell me about.					
yes	20%	47%	26%	40%	*χ2* (6, N = 461) = 13.2, *p* < .05)
no	78%	53%	70%	57%	
I do not know	2%	0%	4%	4%	

*Note*. For reasons of space, the wording of the questions displayed in this table is abbreviated. Please see S1 File for the exact question wording in the original questionnaire. Percentages might not add up to 100% due to rounding errors. All reported *p*-values are Bonferroni corrected.

## Discussion

Based on a large sample, our study sheds some light on preferences surrounding a person’s wish to know or not to know and potentially associated factors. Results from previous surveys of the general population and patients suggest that the overwhelming majority of persons surveyed are in favor of learning about health information and incidental and secondary findings, and also about raw data on their own health condition [20–23,25,28,29,39]. This general tendency has been replicated in our study. However, even though people seem to be interested in learning their results and anticipate positive behavioral change [22], the majority of our participants was also of the opinion that genetic testing has the potential for negative consequences (e.g. psychological distress, societal discrimination).

To study informational preferences in-depth, we varied the characteristics of genomic findings and analyzed how this affects the ‘wish to know’. The finding of previous research that preference for disclosure depends on features of the to-be-diagnosed disease (such as its controllability) [32–34] was replicated in our study. Our results furthermore indicate that the amount of information given about the potential consequences of an incidental finding affected the participants’ wish to know (Fig 1). When the question is phrased in an abstract and reduced way (e.g., “I want to know about any disease I have that is found incidentally”), an overwhelming majority preferred disclosure of the finding. But when it came to scenarios with a detailed description of the finding (e.g. name and symptoms of the disease), the percentage of persons consenting to full disclosure dropped considerably.

Cognitive psychologists have gathered ample evidence that the level of abstraction at which a situation is represented has profound effects on subsequent judgment and decision-making [40]. While an abstract construal leads to a focus on the central features of a choice object (e.g., the main purpose of a medical test, which is to benefit the recipient), peripheral features (such as psychological distress that might arise as unintended side-effect in some concrete diagnostic scenarios) are usually only represented at a more concrete level of construal. This asymmetry has important implications for the clinical practice of informed consent. If patients consent to a genetic test while they represent it in a highly abstract fashion, they might underestimate the extent to which they will be negatively affected when they are actually faced with a concrete disturbing outcome in the future. We should thus ask ourselves if it is enough to ask patients or participants whether they want to receive information on incidental or secondary findings or not. It could be important to mention the possibility of negative consequences. In order to make an educated decision as to whether one wants to receive information about incidental or secondary findings one should have the possibility to reflect about a number of potential concrete scenarios that can arise from the disclosure (e.g. clinical validity, interpretation of risk information, potential psychological distress and discrimination).

A study population of over 500 participants drawn from a diverse background (e.g. healthcare professionals, patients, general population) enabled us to compare the views of different groups. While prior research found that patients want to receive information about incidental findings more than the general population and health care professionals [39], our data revealed no differences between individuals who are personally affected and those who are not. Rather, in our sample the educational level and the professional role in the healthcare system were most strongly associated to the expressed attitudes. Previous findings showed that experts and professionals of the healthcare system tend to be more reflected regarding this topic as they mainly wish to learn results that are actionable [27,30,39]. Our results replicate these findings in that physicians and medical students tend to be more selective in the kind of information they want to receive. The same is true more generally for participants with higher levels of education.

### Limitations and outlook

The hypothetical character of the questionnaire has to be discussed. When being confronted with the questionnaire the participants make a judgment about hypothetical scenarios. Being in an actual decision situation (a pre-counselling session in clinical or research context) might lead to a deeper elaboration of the topic. It should be noted, though, that the judgment about one’s openness towards an incidental or secondary finding in a real clinical scenario is also to some extent hypothetical in character, as the potentially relevant test results do not (yet) exist at the moment of decision. We are therefore confident that our findings have external validity despite their hypothetical character.

Concerning our sample it is important to be aware that the collection of participants who completed the paper-and-pencil version of the questionnaire mainly took place in the context of the Göttingen University Medical Center. The recruitment via online-version was launched nationwide in different newsletters. The sample is not representative for the whole country, so the findings cannot be generalized to all German citizens. Our goal was rather to investigate a sample that is heterogeneous with regards to participants’ involvement with genetic testing (as patient or practitioner, or without any connection to the subject) to allow for an exemplary comparison of these groups’ attitudes towards incidental or secondary findings.

Our results showed that the factors ‘professional role in the healthcare-system’ and ‘educational level’ are associated with these attitudes. Since both factors are likely correlated to each other and to other potentially explanatory variables that we did not measure (e.g., socio-economic status), we cannot conclude from the observed relationships on which of these factors, if any, participants’ attitudes causally depend. Future research is needed to disentangle the causal roles and relative contributions of the different predictors. Such studies would require a more systematic composition of the sample.

In general, this explorative study shows that, while people seem to be generally interested in learning about personalized genetic information, personal factors and the way in which questions are posed can lead to different opinions that might potentially result in different decisions. As a conclusion, these factors should be further examined and then be considered in clinical practice. Clinicians and researchers in the field of genetics have to reflect the way in which they explain the situation (e.g. abstract question vs. concrete scenario), and they might have to take the personal situation and background of the individuals in question into account. The integration of the concept of the ‘right not to know’ with all its ramifications into clinical and research contexts should follow a thoroughly deliberated fashion, as a kind of ‘disclosure before disclosure’ that allows recipients to make responsible use of their right to informational self-determination.

## Supporting information

S1 Dataset Raw data. (XLS)

S1 File Questionnaire. (DOCX)
