# Supplementary material for: Genomic information and a person’s right not to know: A closer look at variations in hypothetical informational preferences in a German sample
Source: PLoS One. 2018 Jun 20;13(6):e0198249. doi: 10.1371/journal.pone.0198249 (PMC6010220; doi:10.1371/journal.pone.0198249)
Supplement: S1 File — (DOCX) [file pone.0198249.s002.docx]

Supplementary Material 1: **Questionnaire**

**Normative foundation and practical validity of the right not to know**

1. Introduction

This questionnaire aims to investigate what information people want to receive about their illnesses and genetic makeup. The research is being performed jointly by lawyers, geneticists, psychiatrists, and ethicists at the universities of Göttingen and Ulm. This research project is being funded by the German Federal Ministry of Education and Research (BMBF). Many illnesses have serious consequences for those affected, whereby the worst consequences often are in the distant future. Frequently, relatives or work colleagues also are affected (genetic disorders, infectious diseases). In other cases, only the person affected becomes ill, but relatives, health insurers, and nursing services have to provide a significant amount of services, perhaps also only in the distant future. In the case of genetic disorders, relatives can get the same disorder or the genes can be passed on. This is true for hemophilia, for example. In the foreseeable future, it will be possible to study a person’s complete genetic makeup and determine all the differences between people. Such differences may not be relevant for the physical and mental health of a person, but they can also cause genetic disorders or diseases with a hereditary component. Depending on the type of genetic disorder, the disease can occur in the unborn child or during childhood, adolescence, or adulthood. Each person has to determine the severity of a genetic disorder for himself during a genetic or psychotherapeutic consultation, taking into consideration his own symptoms, the symptoms of affected relatives, and the information about the prognosis and potential treatments. The following questionnaire aims to investigate whether, to what extent, and at what time people want to know what they or their environment has to expect, e.g. if they are carrying alterations in their genetic makeup. Lawyers, physicians, and ethicists disagree about what a person wants or is allowed to or even must know. You will help us greatly by completing this questionnaire. The responses should reflect your personal attitude, therefore there are no “right” or “wrong” answers. The survey is anonymous, all person-related data will be deleted when the results are analyzed. Your participation is voluntary, and you can discontinue at any time.

Thank you for participating.

1. The Right Not to Know
2. Everybody has the right to know everything about his genetic makeup and also about the risk for genetic disorders.

- Completely not agree – completely agree
- Don´t know

1. Genetic tests can result in people who are found to have a genetic disorder being socially discriminated or excluded from society.

- Completely not agree – completely agree
- Don´t know

1. In research studies (clinical studies and other research studies), a future participant has to be completely informed about the risks and benefits before he can agree to participate (“informed consent”). Do you agree with this legal provision? (Multiple responses possible)

- Yes, completely agree
- Yes, but he should also be able to choose not to be informed
- Yes, but only if the participant understands the information
- No, I think all that giving of information is over the top
- No, the information given is often so bad that it can make you ill
- Don´t know

1. I want to know about any disease I have that is found incidentally.

- Yes (1), No (2), Only, if prevention possibilities are available (3)
- Don´t know (4)

1. I want to know about any genetic disorder I have that is found incidentally.

- Yes (1), No (2), Only, if prevention possibilities are available (3)
- Don´t know (4)

1. I want to know about any risk I have for a genetic disorder that is found incidentally.

- Yes (1), No (2), Only, if prevention possibilities are available (3)
- Don´t know (4)

1. There is a simple and reasonably priced option to be tested for your risk for more than 250 genetic disorders. Would you get yourself tested?

- Completely not agree – completely agree
- Don´t know

1. Before a test or study during which something could be found incidentally, I’d like to discuss with my physician in detail about what I do and do not want to know, even if the conversation would take a very long time.

- Yes, in as much detail as possible
- I’d like to express my wishes a little, but my physician should make most of the decisions for me
- My physician should decide himself what he does and doesn’t tell me
- I don’t want to know anything at all about possible incidental findings
- Don’t know

1. What conditions are important for the decision whether your physician should or should not tell you about a finding regarding an illness?

(Multiple responses possible)

- I always want to be informed about such a finding.
- Depends on the severity
- Depends on whether it’s treatable
- Depends on the inheritability
- I never want to be informed about such a finding
- I don’t have an opinion about this question

1. What conditions are important regarding the question of whether your physician should or should not tell you if a risk is found?

- Depends on the severity of the possible illness
- Depends on the certainty/uncertainty of the illness occurring
- Depends whether the illness will occur in the near or distant future
- Depends on the preventive options
- Depends on whether it’s treatable
- Depends on the inheritability
- I always want to be informed about such a finding.
- I never want to be informed about such a finding
- I don’t have an opinion about this question

1. Do you think that the knowledge about having a high risk for a fatal, non-treatable illness would be a burden in your life?

- Completely not agree – completely agree
- Don´t know

1. During a routine exam, your physician discovers that you have a serious illness. There are treatment options. What do you want to know? (Multiple responses possible)
   - My physician should decide what he thinks is important for me
   - Name of the illness
   - What I can do to halt the progress of/alleviate the illness
   - Probable course
   - Don’t know
2. During a routine exam, your physician discovers that you have a serious illness. There are no treatment options. What do you want to know?
   - My physician should decide what he thinks is important for me
   - Name of the illness
   - What I can do to halt the progress of/alleviate the illness
   - Probable course
   - Possibility of heredity
   - Probable remaining lifespan
   - Don´t know
3. Imagine that you are 18 years old. You know that a severe, non-treatable genetic disorder occurs in your family. You could have inherited the responsible gene defect. The age of onset of the illness differs from family to family. Depending on the following age of onset of the illness, would you want to know if you have the genetic defect?
   1. Age of onset of illness approx. 20 years

- Completely not agree – completely agree
- Don´t know
  1. Age of onset of illness approx. 40 years
- Completely not agree – completely agree
- Don´t know
  1. Age of onset of illness approx. 60 years
- Completely not agree – completely agree
- Don´t know

1. During a research study, you are found by chance to have chronic hepatitis C. However, you don’t have any symptoms. If it is not treated, the illness has a 50% chance of leading to death in 10-30 years. Chronic hepatitis C can usually be successfully treated. The treatment takes a long time and has side effects. Until you are cured, you will remain infectious. The illness is transmitted through sex, blood exchange (blood donation, sharing toothbrushes), and perhaps also through living close together. What do you want?
   - No information, I have a right not to know
   - Just brief information and a copy of the finding for my family practitioner
   - Information about the reporting requirements and behavioral measures
   - Immediate, complete information and referral to an experienced specialist
   - Information by telephone/in writing also to my partner
   - Don´t know
2. During a study, it is discovered incidentally that you have a tendency to develop Alzheimer’s dementia, which has a 50% chance of appearing in approx. 10 years. At the moment, you’re healthy and feel well. If you do develop Alzheimer’s dementia, you will experience increasing memory loss and then die of the Alzheimer’s dementia within 10 years. What do you want if this happens?
   - Information if an “abnormality” is determined, with the option to have time to consider before receiving further information
   - Brief information and immediate referral to family practitioner/specialist
   - No information, I have a right not to know
   - Just brief information and a copy of the finding for my family practitioner
   - Don´t know
3. Imagine you had severe depression for 9 months. The illness completely disappeared with treatment, but it can reoccur. Your family practitioner estimates the risk of a reoccurrence at 50%. However, you will be healthy for at least 5 years. What do you want to know?
   - No information, I want to live carefree
   - I want to know something only if I can prevent it reoccurring
   - I want to know exactly whether and under what conditions the illness can reoccur
   - Don´t know
4. Several members of your family have died of a heart attack. The risk for cardiovascular disease has a hereditary component, but it can be influenced by a healthy diet, exercise, and weight control. Do you want to know if you inherited the genetic predisposition for this illness?

- Completely not agree – completely agree
- Don´t know

1. Several members of your family have hereditary breast cancer. If you have inherited the genetic predisposition for breast cancer, there are certain precautionary measures you can take. Do you want to know if you have the genetic predisposition?

- Completely not agree – completely agree
- Don´t know

1. Several members of your family have hereditary cancer for which there are no precautionary measures you can take. Do you want to know if you have a risk (greater than 50%) of getting one of those cancers?

- Completely not agree – completely agree
- Don´t know

1. Several people in your family have been reliant on a wheelchair since the age of 30-40 because of a genetic disorder that causes muscular atrophy. Do you want to know if you have inherited the genetic makeup for muscular atrophy and will need a wheelchair at age approx. 30-40 years?

- Completely not agree – completely agree
- Don´t know

1. Patient-Doctor-Relationship
2. My physician should know all my genetic findings and decide on the basis of his professional knowledge which he tells me about and which he doesn’t tell me about.

- Completely not agree – completely agree
- Don´t know

1. Do you think that as a patient or participant in a study you should always be informed about incidental findings?

- Completely not agree – completely agree
- Don´t know

1. Do you think that a doctor can ignore a patient’s wishes concerning receiving information about illness/risks?
   - Yes, always, because of his greater expertise
   - No, never. I always have to be able to decide myself what I want to know and what not.
   - Only in exceptional cases
   - Don´t know
2. Which of the following do you think outweighs the other: The physician’s duty of care towards you as a patient or your right to self-determination, to decide yourself what you want to know about yourself and what not?
   - Don´t know
   - Duty of care
   - Right to self-determination
3. You refused to be told about incidental findings. However, your physician thinks it’s important that you are informed about an incidental finding. He thinks he has the responsibility and the right to inform you about it,
   1. If you have a **disease** for which there are good treatment options.
      - I agree
      - I reject being given information against my will
      - Don´t know
4. If you have a fatal **disease** for which there are no treatment options.
   - - I agree
     - I reject being given information against my will
     - Don´t know
5. If you have a **risk** for a fatal disease for which there are good treatment options.
   - - I agree
     - I reject being given information against my will
     - Don´t know
6. If you have a **risk** for a fatal disease for which there are no treatment options.
   - - I agree
     - I reject being given information against my will
     - Don´t know
7. You previously refused to be informed about incidental findings. However, your physician thinks it’s important that you are told you have a **disease**. He thinks he has the responsibility and the right to tell you about it.
   - - I agree
     - I reject being given information against my will
     - Don´t know
8. You previously refused to be informed about incidental findings. However, your physician thinks it’s important that you are told you have a **high risk** for a fatal disease. There are no treatment options. He thinks he has the responsibility and the right to tell you about it.
   - - I agree
     - I reject being given information against my will
     - Don´t know
9. You previously refused to be informed about incidental findings. However, your physician thinks it’s important that you are informed you have a **risk** for a disease, because there are good precautionary measures you can take. He thinks he has the responsibility and the right to tell you about it.
   - - I agree
     - I reject being given information against my will
     - Don´t know
10. Sharing information with a third party

Because to a certain extent your relatives have the same genetic makeup as you, they may want to know about your genetic disorders or risks for genetic disorders. In the case of infectious diseases, you can infect also other people if you don’t take precautionary measures. But to do so, you have to know what pathogens you are carrying and whether you’re infectious. Employers and insurers could also have great (economic) interest in some of your genetic information or in information about whether you have an infectious disease.

1. Imagine that you have a genetic test performed. The results could be highly relevant for the physical and mental health of your relatives. Do you tell your relatives about your test?
   - - Yes, before the test is performed
     - Yes, but only after the test
     - Don´t know
2. You are found to have genetic disorder or a risk of disease that may also affect your relatives. Do you want them also to be informed about it?
   - - Yes, by the family practitioner
     - Yes, but I should tell them myself
     - No, my relatives shouldn’t be given any information.
     - Don´t know
3. Should relatives have the right not to receive information about the genetic disorder or about the risk for the genetic disorder?

- Completely not agree – completely agree
- Don´t know

1. A genetic test is performed on one of your relatives. If the result affects also you, do you want to be informed about it?

- Completely not agree – completely agree
- Don´t know

1. Imagine your child wants to have a genetic test for a genetic disorder that your own mother died of. If the test finds that your child will get the disorder, then you will definitely get it, too. But you don’t want to know that. Should your child be refused the test?

- Completely not agree – completely agree
- Don´t know

1. Should various insurances (life insurance, occupational disability insurance, disability insurance, long-term care insurance) have the right to have their applicants/members tested for a genetic risk, to allow them perhaps to adjust the contribution amount according to the determined risk?

- Completely not agree – completely agree
- Don´t know

1. Should people who have jobs with special responsibility (e.g. pilots) be tested for certain genetic risks?

- Completely not agree – completely agree
- Don´t know

1. Imagine you had a life partner with whom you are planning a longer-term partnership. Would you advise your partner to be tested for his or her genetic risk for a genetic disorder?

- Yes, so that he/she can inform himself
- Yes, so that I find out what risks exist
- Yes, so that—depending on the result—I can end the partnership
- Yes, because of the risk for (future) children
- No, a test isn’t important to me
- Don´t know

1. Would you have yourself genetically tested so you can better assess the risk that (future) children will develop a serious disease?

- Completely not agree – completely agree
- Don´t know

1. Would you want to know already before the birth whether your child has a genetic risk for a genetic disorder?

- Completely not agree – completely agree
- Don´t know

1. You are found to have a genetic defect that you can pass on to your children but that cannot cause a disease in you. Do you want to be informed about it?

- Completely not agree – completely agree
- Don´t know
